# Reference ranges of fetal superior vena cava blood flow velocities and pulsatility index in the second half of pregnancy: a longitudinal study

**DOI:** 10.1186/s12884-021-03635-6

**Published:** 2021-02-23

**Authors:** Maria Stefopoulou, Lotta Herling, Jonas Johnson, Peter Lindgren, Torvid Kiserud, Ganesh Acharya

**Affiliations:** 1 Division of Obstetrics and Gynecology, Department of Clinical Science, Intervention and Technology (CLINTEC), Karolinska Institutet and Center for Fetal Medicine Karolinska University Hospital, 14186 Stockholm, Sweden; 2 Women’s Health and Perinatology Research Group, Department of Clinical Medicine, Faculty of Health Sciences, UiT-The Arctic University of Norway, Tromsø, Norway; 3 Department of Obstetrics and Gynecology, University Hospital of North Norway, Tromsø, Norway; 4 Department of Clinical Science, University of Bergen, Bergen, Norway; 5 Department of Obstetrics and Gynecology, Haukeland University Hospital, Bergen, Norway

**Keywords:** Blood flow velocity, Fetus, hemodynamics, Pulsatility index, Reference ranges, superior vena cava, Venous Doppler

## Abstract

**Background:**

Fetal superior vena cava (SVC) is essentially the single vessel returning blood from the upper body to the heart. With approximately 80-85% of SVC blood flow representing cerebral venous return, its interrogation may provide clinically relevant information about fetal brain circulation. However, normal reference values for fetal SVC Doppler velocities and pulsatility index are lacking. Our aim was to establish longitudinal reference intervals for blood flow velocities and pulsatility index of the SVC during the second half of pregnancy.

**Methods:**

This was a prospective study of low-risk singleton pregnancies. Serial Doppler examinations were performed approximately every 4 weeks to obtain fetal SVC blood velocity waveforms during 20–41 weeks. Peak systolic (S) velocity, diastolic (D) velocity, time-averaged maximum velocity (TAMxV), time-averaged intensity-weighted mean velocity (TAMeanV), and end-diastolic velocity during atrial contraction (A-velocity) were measured. Pulsatility index for vein (PIV) was calculated.

**Results:**

SVC blood flow velocities were successfully recorded in the 134 fetuses yielding 510 sets of observations. The velocities increased significantly with advancing gestation: mean S-velocity increased from 24.0 to 39.8 cm/s, D-velocity from 13.0 to 19.0 cm/s, and A-velocity from 4.8 to 7.1 cm/s. Mean TAMxV increased from 12.7 to 23.1 cm/s, and TAMeanV from 6.9 to 11.2 cm/s. The PIV remained stable at 1.5 throughout the second half of pregnancy.

**Conclusions:**

Longitudinal reference intervals of SVC blood flow velocities and PIV were established for the second half of pregnancy. The SVC velocities increased with advancing gestation, while the PIV remained stable from 20 weeks to term.

## Background

Assessment of fetal well-being is important in obstetrics for optimizing clinical management, and for that purpose, Doppler ultrasonography is one of the most commonly used methods [1]. It has the capacity to detect changes in the hemodynamics of compromised fetuses [2, 3]. In case of hypoxemia, such fetuses are known to redistribute blood to vital organs, i.e. brain, heart and adrenal glands [4, 5]. On the arterial side, Doppler velocimetry of the umbilical and middle cerebral arteries is often used to identify this phenomenon [6]. On the venous side, the ductus venosus and umbilical vein are the most commonly used for the evaluation of fetal wellbeing [7, 8]. Fetal inferior vena cava (IVC) flow patterns have also been studied [9, 10], but these veins represent the venous return from the lower body and the placenta. On the other hand, the superior vena cava (SVC) is essentially the single vessel returning blood to the heart from the head and upper body. As approximately 80% of SVC blood flow comes from the brain [11], its interrogation may provide clinically useful information. In preterm neonates, SVC blood flow measurement has been shown to be a useful tool in assessing hemodynamics and predicting complications, e.g. cerebral intraventricular hemorrhage [12]. However, this compartment of the venous circulation has not been studied extensively in the fetus.

In 1990, Reed et al. described the blood flow velocity waveform patterns of the SVC and IVC in normal and growth restricted fetuses, and in fetuses with cardiac arrhythmia demonstrating that the time-velocity integral of diastolic waveform was lower in severly growth restricted fetuses, and that the reversed end-diastolic velocity was augmented during atrial contraction in fetal tachyarrythmias [13]. A decade later, Fouron et al. reported a similar study comparing SVC and IVC Doppler velocity waveform profiles between 15 normally grown fetuses and 11 fetuses with absent end-diastolic flow in the umbilical artery showing that the SVC waveforms resemble IVC waveforms and vice versa in growth-restricted fetuses with severe placental insufficiency [14]. In 2012, Nyberg et al. showed that the SVC blood flow increased significantly during fetal breathing movements in normal pregnancy [15]. In this publication they also presented baseline normative data on peak systolic (S) velocity and time-averaged maximum velocity (TAMxV) based on a total of 302 measurements obtained from 110 fetuses at 3 gestational age windows. However, their study and design were not aimed at establishing gestational age specific reference values for SVC velocities nor was the pulsatility index for veins (PIV) addressed.

Accordingly, our aim for the present study was to establish longitudinal reference intervals for the fetal SVC blood velocities and PIV for the second half of pregnancy.

## Methods

### Study population

This was part of a prospective observational study on fetal cardiovacular function conducted at the Department of Obstetrics and Gynecology, University Hospital of North Norway, Tromsø, Norway, during 2009–2012. Pregnant women were informed about the study and recruited at the time of routine ultrasound screening at 18–20 gestational weeks. A total of 142 women with uncomplicated singleton pregnancies were enrolled in the study after obtaining written consent, and examined approximately every 4 weeks until term. Some data on impedance based and volume blood flow based cerebro-placental and umbilico-cerebral ratios from this study population have been reported previously [16, 17].

All participants had their routine second trimester scan when their gestational age was confirmed by the measurement of biparietal diameter or head circumference. Inclusion criteria were: age ≥ 18 years, uncomplicated singleton pregnancy, and gestational age ≥ 18 and < 24 weeks at enrollment. Exclusion criteria were: preeclampsia in previous pregnancy, gestational diabetes or history of preexisting maternal diseases complicating the pregnancy, such as chronic hypertension, diabetes mellitus or autoimmune disease. Fetuses with growth restriction (defined as estimated fetal weight < 10th percentile and umbilical artery pulsatility index >95th percentile for the gestational age), major congenital malformations or chromosomal abnormality were also excluded.

### Doppler ultrasonography

The ultrasound examinations were performed transabdominally with the pregnant woman in a supine semirecumbent position. Three specialist obstetricinas experienced in ultrasonography performed the Doppler ultrasonographic measurements. Ultrasonography was performed using a Vivid 7 Dimension ultrasound system (GE Vingmed Ultrasound AS, Horten, Norway) equipped with a M4S sector transducer with frequencies of 1.5–4.3 MHz. At each study visit, after confirming the presence of fetal heart activity, fetal biometry was perfomed to estimate the fetal weight and umbilical artery and middle cerebral artery (MCA) Doppler pulsatility indices were measured as reported previously [16, 17].

The SVC was assessed in a long axis view where it enters the right atrium. The Doppler sample gate was placed at the point where the vessel enters the right atrium without overlapping the ascending aorta, using the method described previously [15, 17]. Velocimetry was performed using color directed pulsed-wave Doppler during fetal quiescence. The scale (pulse repetition frequency) was adjusted according to velocities to avoid aliasing. A large enough sample volume was used for Doppler interrogation depending on the gestational age of the fetus and size of the SVC to ensure sampling of the velocities from the entire lumen of the vessel. Doppler insonation was aligned to SVC flow direction. When complete alignment was not possible, angle correction was used to reduce the error in velocity measurement, but never exceeded 30 degrees. The wall motion filter was set to less than 100 Hz. The 3–6 s of cine loop of Doppler velocity waveforms were acquired at a sweep speed of 50–100 cm/s and stored for off-line analysis.

**Fig. 1:**
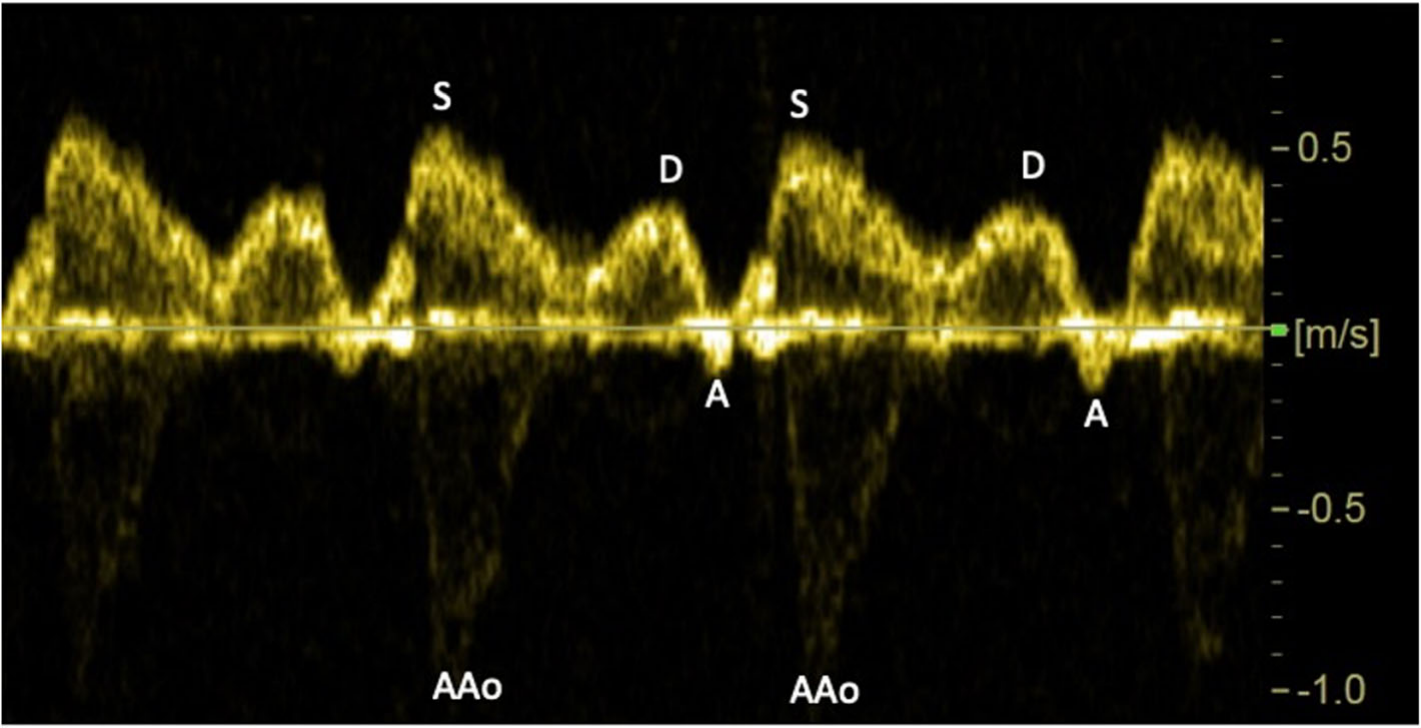
Doppler recording of the fetal superior vena cava blood velocity waveforms showing systolic peak velocity (S), diastolic peak velocity (D), and velocity deflection during atrial contraction (A). Interference by the systolic peak in the ascending aorta (AAo) seen below the zero line

The maximum velocity envelope of the SVC waveforms was automatically traced using the software of the ultrasound machine. The trace was visually inspected and sensitivity adjusted if required. Manual tracing was used if automated tracing was not appropriate. The peak systolic (S) velocity, diastolic (D) velocity, time-averaged maximum velocity (TAMxV), time-averaged intensity weighted mean velocity (TAMeanV) and end-diastolic velocity during the atrial contraction (A-velocity) were measured (Fig. 1). An average value of at least three consecutive cardiac cycles were recorded for analysis. The PIV was calculated as: (S-velocity – A-velocity)/TAMxV as described for other precordial veins [18, 19].

### Clinical outcomes

All pregnant women participating in the study had regular antenatal follow-up according to clinical routine. Data on the course of pregnancy, birth, and neonatal outcome were collected from the electronic medical records.

#### Statistical analysis

The sample size was calculated based on the formula proposed by Royston and Altman according to which the sample size of a longitudinal study is equivalent to the number of participants required to construct gestational age-specific reference ranges in a cross-sectional study divided by a design factor D (i.e. the number of fetuses in a cross-sectional study that would give the same precision as one fetus in a longitudinal study) that equals to 2.3 [20, 21]. Assuming that approximately 15 observations per gestational week (a total of 300 pregnancies from 20 to 40 gestational week), are required in a cross-sectional study to construct gestational age specific reference intervals with adequate precision, we calculated the number of fetuses required for this longitudinal study to be 300/2.3 = 130. We aimed to enroll approximately 140 pregnant women to compensate for possible unsucessful measurements, dropouts, and loss of follow ups.

Data analysis was performed using IBM SPSS Statistics for Windows. Version 24.0. (IBM Corp. Armonk. NY) and MATLAB R2019a (Matworks. Inc. Natick. MA). Data distribution for normality was checked by using the Shapiro-Wilk test. Logarithmic or power transformations were used to achieve normal distribution of the data. The best transformation was chosen based on the Box-Cox transformation lambda values (λ) calculated for each dependent variable. All SVC velocities were log_10_ transformed except the A-velocity that required square (^∧^2) transformation, and the SVC PIV needed 1/SQRT transformation. Best fitting fractional polynomials were chosen to construct gestational age specific mean curves for each variable from a list of 44 regression models based on R^2^ value. Multilevel regression modeling was used to calculate the mean and percentiles of SVC velocities and PIV in relation to gestational age taking into account the repeated measures design of the study [22]. Association between variables were tested using mean vector for each variable from the mixed models. A *P*-value < 0.05 was considered as statistically significant.

## Results

Of the 142 low-risk pregnant woman enrolled, one was excluded from the study due to missing follow-up data, and another seven because SVC Doppler was not recorded. This resulted in a study population of 134 women and a total of 575 obervations with 510 (88.7%) successful recordings of adequate quality SVC blood flow velocity waveforms. The baseline characteristics of the study population and pregnancy outcomes are presented in Table 1. There were no perinatal deaths, but one neonate delivered at 32^+ 4^ weeeks by emergency cesarean section due to fetal distress associated with abruptio placenta, had intraventricular bleeding leading to hydrocephalus requiring shunting. All other babies were healthy on discharge from the hospital.

**Table 1:** Baseline characteristics of the study population and pregnancy outcomes (*n* = 134)

Variable	Median (range), Mean (SD) or n%
Maternal age (years)	30 (19–39)
Maternal body-mass index (Kg/cm^2^)	23.90 (3.80)
Nullipara	61 (45.5%)
Gestational age at birth (weeks)	40 (33–42)
Preterm delivery	
< 37 weeks	4 (3.0%)
< 34 weeks	1 (0.7%)
Pre-eclampsia	4 (3.0%)
Gestational diabetes	1 (0.7%)
Induction of labor	17 (12.7%)
Mode of delivery	
Normal vaginal delivery	116 (86.6%)
Operative vaginal delivery	1 (0.7%)
Cesarean section	17 (12.7%)
Birthweight (g)	3600 (2251–4636)
Sex (male/female)	74 (55) /60 (45)
Apgar score < 7 at 1 min	6 (2.2%)
Apgar score < 7 at 5 min	2 (1.4%)
Admission to neonatal inensive care	5 (3.7%)

Data are presented as median (range). mean (SD) or n (%) as appropriate

All the SVC velocities were postive (antegrade), but the A-velocity was zero in 5.7% and negative (retrograde) in 8.9% of observations. The mean fetal heart rate was 137–140 beats/min.

Gestational age-specific reference values for each SVC velocity variables and PIV with their corresponding 2.5th, 5th, 10th, 50th, 90th, 95th and 97.5th percentiles, are presented in Tables 2, 3, 4, 5, 6, 7.

**Table 2:** Percentiles for fetal superior vena cava peak systolic velocity at 20–40 weeks of gestational age (GA)

		Percentile						
GA (weeks)	n	2.5th	5th	10th	50th	90th	95th	97.5th
20	21	15.07	16.13	17.44	23.00	30.34	32.81	35.12
21	33	15.40	16.51	17.88	23.71	31.43	34.04	36.49
22	30	15.75	16.90	18.33	24.43	32.56	35.32	37.90
23	32	16.10	17.30	18.79	25.17	33.73	36.64	39.37
24	17	16.46	17.70	19.26	25.94	34.94	38.01	40.90
25	30	16.82	18.12	19.75	26.73	36.19	39.43	42.48
26	29	17.20	18.55	20.24	27.55	37.49	40.91	44.13
27	27	17.58	18.99	20.75	28.39	38.84	42.44	45.84
28	27	17.97	19.44	21.27	29.25	40.23	44.03	47.62
29	21	18.37	19.90	21.81	30.15	41.68	45.68	49.46
30	35	18.78	20.37	22.35	31.07	43.17	47.39	51.38
31	27	19.20	20.85	22.92	32.01	44.72	49.16	53.37
32	19	19.63	21.34	23.49	32.99	46.33	51.00	55.44
33	25	20.07	21.84	24.08	34.00	47.99	52.91	57.59
34	25	20.52	22.36	24.69	35.03	49.71	54.89	59.82
35	27	20.97	22.89	25.31	36.10	51.50	56.95	62.14
36	20	21.44	23.43	25.94	37.20	53.35	59.08	64.55
37	20	21.92	23.98	26.60	38.34	55.26	61.29	67.05
38	25	22.41	24.55	27.27	39.51	57.25	63.59	69.65
39	18	22.91	25.13	27.95	40.71	59.30	65.97	72.35
40	2	23.42	25.72	28.65	41.95	61.43	68.44	75.16

**Table 3:** Percentiles for superior vena cava diastolic (D)-velocity at 20–40 weeks of gestational age (GA)

		Percentile						
GA (weeks)	n	2.5th	5th	10th	50th	90th	95th	97.5th
20	21	7.78	8.45	9.30	13.04	18.28	20.12	21.86
21	31	7.83	8.53	9.40	13.29	18.78	20.71	22.55
22	30	7.89	8.60	9.51	13.54	19.29	21.32	23.26
23	31	7.94	8.68	9.62	13.81	19.82	21.95	23.99
24	17	8.00	8.76	9.73	14.07	20.36	22.60	24.75
25	30	8.06	8.84	9.84	14.34	20.91	23.27	25.53
26	29	8.12	8.92	9.95	14.62	21.48	23.95	26.33
27	27	8.17	9.00	10.06	14.90	22.06	24.66	27.16
28	26	8.23	9.08	10.18	15.19	22.66	25.39	28.01
29	21	8.29	9.17	10.29	15.48	23.28	26.13	28.89
30	35	8.35	9.25	10.41	15.78	23.92	26.91	29.80
31	27	8.41	9.34	10.53	16.08	24.57	27.70	30.74
32	19	8.47	9.42	10.64	16.39	25.24	28.52	31.71
33	25	8.53	9.51	10.77	16.71	25.92	29.36	32.70
34	25	8.59	9.59	10.89	17.03	26.63	30.22	33.73
35	26	8.66	9.68	11.01	17.35	27.35	31.11	34.79
36	20	8.72	9.77	11.14	17.69	28.10	32.03	35.89
37	20	8.78	9.86	11.26	18.03	28.86	32.98	37.02
38	25	8.84	9.95	11.39	18.38	29.65	33.95	38.18
39	18	8.91	10.04	11.52	18.73	30.45	34.95	39.38
40	2	8.97	10.13	11.65	19.09	31.28	35.98	40.62

**Table 4:** Percentiles for superior vena cava time-averaged maximum velocity (TAMxV) at 20–40 weeks of gestational age (GA)

		Percentile						
GA (weeks)	n	2.5th	5th	10th	50th	90th	95th	97.5th
20	21	8.20	8.81	9.56	12.76	17.03	18.48	19.84
21	33	8.40	9.02	9.81	13.14	17.62	19.15	20.58
22	30	8.60	9.25	10.06	13.54	18.23	19.83	21.34
23	32	8.80	9.48	10.32	13.95	18.87	20.55	22.13
24	17	9.01	9.71	10.59	14.38	19.52	21.29	22.95
25	30	9.22	9.95	10.87	14.81	20.20	22.05	23.79
26	29	9.44	10.20	11.15	15.26	20.90	22.84	24.68
27	27	9.67	10.45	11.44	15.73	21.62	23.66	25.59
28	27	9.89	10.71	11.74	16.20	22.37	24.51	26.54
29	21	10.13	10.98	12.04	16.70	23.15	25.39	27.52
30	35	10.37	11.25	12.35	17.20	23.95	26.31	28.54
31	27	10.61	11.53	12.67	17.72	24.78	27.25	29.59
32	1 9	10.87	11.81	13.00	18.26	25.64	28.23	30.69
33	25	11.12	12.10	13.34	18.82	26.53	29.25	31.82
34	25	11.39	12.40	13.69	19.39	27.45	30.30	33.00
35	27	11.66	12.71	14.04	19.97	28.41	31.39	34.22
36	20	11.93	13.03	14.41	20.58	29.39	32.51	35.49
37	20	12.22	13.35	14.78	21.20	30.41	33.68	36.80
38	25	12.51	13.68	15.17	21.85	31.47	34.89	38.17
39	18	12.80	14.02	15.56	22.51	32.56	36.15	39.58
40	2	13.11	14.37	15.97	23.19	33.69	37.45	41.04

**Table 5:** Percentiles for superior vena cava time-averaged intensity weighted mean velocity (TAMeanV) at 20–40 weeks of gestational age (GA)

		Percentile						
GA (weeks)	n	2.5th	5th	10th	50th	90th	95th	97.5th
20	19	4.43	4.77	5.20	7.02	9.47	10.31	11.10
21	32	4.48	4.83	5.27	7.18	9.79	10.68	11.52
22	30	4.53	4.89	5.35	7.36	10.11	11.06	11.96
23	32	4.57	4.95	5.43	7.53	10.44	11.45	12.41
24	17	4.62	5.02	5.52	7.71	10.79	11.86	12.88
25	29	4.67	5.08	5.60	7.90	11.14	12.28	13.36
26	29	4.72	5.14	5.68	8.09	11.51	12.72	13.87
27	27	4.77	5.21	5.77	8.28	11.89	13.17	14.39
28	24	4.81	5.27	5.86	8.48	12.28	13.64	14.94
29	20	4.86	5.34	5.94	8.68	12.68	14.12	15.50
30	34	4.91	5.41	6.03	8.89	13.10	14.62	16.09
31	25	4.97	5.47	6.12	9.10	13.54	15.14	16.69
32	17	5.02	5.54	6.22	9.32	13.98	15.68	17.32
33	24	5.07	5.61	6.31	9.55	14.44	16.24	17.98
34	24	5.12	5.68	6.41	9.78	14.92	16.82	18.66
35	27	5.17	5.75	6.50	10.01	15.41	17.41	19.36
36	18	5.23	5.83	6.60	10.25	15.92	18.03	20.09
37	19	5.28	5.90	6.70	10.50	16.44	18.67	20.85
38	25	5.34	5.97	6.80	10.75	16.99	19.34	21.64
39	17	5.39	6.05	6.90	11.00	17.55	20.02	22.46
40	2	5.45	6.12	7.01	11.27	18.12	20.74	23.30

**Table 6:** Percentiles for superior vena cava end-diastolic velocity during atrial contraction (A-velocity) at 20–40 weeks of gestational age (GA)

		Percentile						
GA (weeks)	n	2.5th	5th	10th	50th	90th	95th	97.5th
20	21	−5.17	−3.05	−0.96	4.83	9.37	10.51	11.47
21	33	−5.32	−3.13	−0.98	4.96	9.58	10.75	11.72
22	30	−5.47	−3.20	−0.99	5.08	9.79	10.98	11.97
23	32	−5.63	−3.28	− 1.00	5.20	10.00	11.21	12.21
24	17	−5.79	−3.35	− 1.01	5.33	10.21	11.43	12.46
25	30	−5.94	−3.43	−1.03	5.45	10.41	11.66	12.70
26	29	−6.11	−3.51	− 1.04	5.57	10.62	11.88	12.94
27	27	−6.27	−3.58	− 1.05	5.69	10.82	12.10	13.17
28	27	−6.44	−3.66	−1.06	5.81	11.02	12.32	13.41
29	20	−6.61	−3.74	−1.08	5.93	11.22	12.54	13.64
30	35	−6.78	−3.82	−1.09	6.05	11.42	12.76	13.88
31	27	−6.96	−3.90	−1.10	6.16	11.61	12.97	14.11
32	19	−7.14	−3.98	−1.11	6.28	11.81	13.19	14.33
33	25	−7.32	−4.06	−1.13	6.40	12.00	13.40	14.56
34	25	−7.51	−4.14	−1.14	6.51	12.20	13.61	14.79
35	27	−7.70	− 4.22	−1.15	6.63	12.39	13.82	15.01
36	20	−7.89	−4.30	−1.17	6.74	12.58	14.03	15.23
37	20	−8.09	−4.38	−1.18	6.86	12.77	14.23	15.45
38	25	−8.30	−4.46	−1.19	6.97	12.95	14.44	15.67
39	18	−8.51	−4.55	−1.20	7.08	13.14	14.64	15.89
40	2	−8.72	−4.63	−1.22	7.20	13.33	14.84	16.11

**Table 7:** Percentiles for superior vena cava pulsatility index for vein (PIV) at 20–40 weeks of gestational age (GA)

		Percentile						
GA (weeks)	n	2.5th	5th	10th	50th	90th	95th	97.5th
20	21	0.93	1.00	1.08	1.49	2.17	2.45	2.74
21	33	0.93	1.00	1.08	1.49	2.17	2.44	2.73
22	30	0.94	1.00	1.09	1.49	2.16	2.44	2.72
23	32	0.94	1.00	1.09	1.49	2.16	2.43	2.72
24	17	0.94	1.01	1.09	1.49	2.16	2.43	2.71
25	30	0.94	1.01	1.09	1.49	2.16	2.43	2.71
26	29	0.95	1.01	1.09	1.49	2.16	2.43	2.70
27	27	0.95	1.01	1.10	1.49	2.16	2.42	2.70
28	26	0.95	1.02	1.10	1.50	2.15	2.42	2.69
29	21	0.95	1.02	1.10	1.50	2.15	2.42	2.69
30	35	0.96	1.02	1.11	1.50	2.15	2.42	2.69
31	27	0.96	1.02	1.11	1.50	2.15	2.42	2.68
32	18	0.96	1.03	1.11	1.51	2.15	2.42	2.68
33	25	0.97	1.03	1.11	1.51	2.16	2.42	2.68
34	25	0.97	1.03	1.12	1.51	2.16	2.41	2.68
35	27	0.97	1.04	1.12	1.51	2.16	2.41	2.68
36	20	0.98	1.04	1.12	1.52	2.16	2.42	2.68
37	20	0.98	1.04	1.13	1.52	2.16	2.42	2.68
38	25	0.98	1.05	1.13	1.52	2.16	2.42	2.68
39	18	0.99	1.05	1.14	1.53	2.16	2.42	2.68
40	2	0.99	1.06	1.14	1.53	2.17	2.42	2.68

**Fig. 2:**
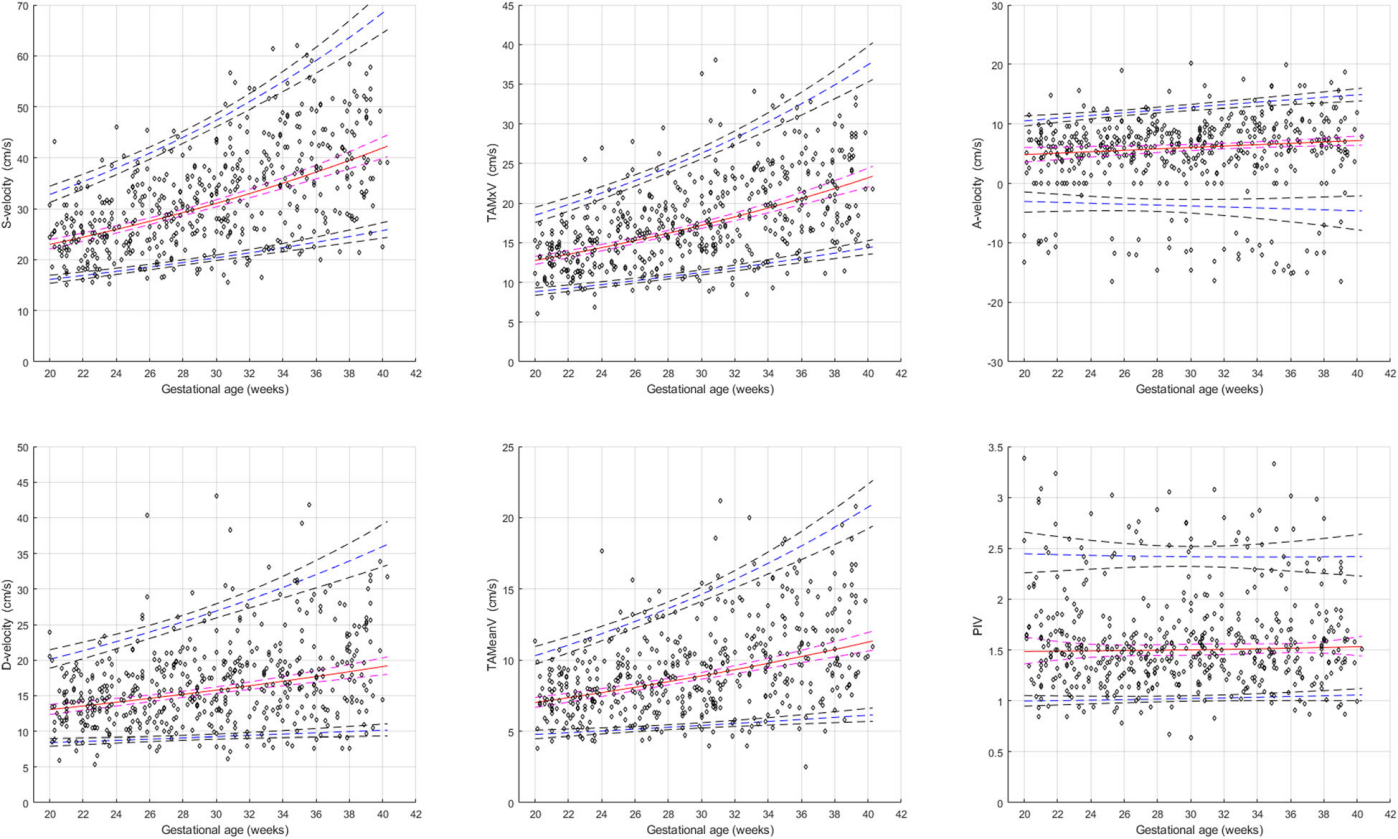
Longitudinal reference ranges for the fetal superior vena cava (SVC) blood flow velocities and pulsatility index for vein (PIV) based on more than 500 individual observations (open circles) in 134 pregnancies, presented as mean (red line) with 95% confidence intervals (interrupted red lines) and 5th and 95th percentiles (interrupted blue lines) with corresponding 95% confidence intervals (interrupted grey lines). Peak systolic (S), diastolic (D), TAMeanV (time-averaged intensity weighted mean velocity), TAMxV (time-averaged maximum velocity), end-diastolic velocity during artrial contraction (A-velocity)

The mean peak S-velocity increased with advancing gestation from 24.0 cm/s at 20 weeks to 39.9 cm/s at 40 weeks, D-velocity from 13.0 at 20 weeks to 19.1 cm/s at 40 weeks, TAMxV from 12.7 cm/s at 20 weeks to 23.2 cm/s at 40 weeks, TAMeanV from 7.0 cm/s at 20 weeks to 11.3 cm/s at 40 weeks, and A-velocity from 4.8 cm/s at 20 weeks to 7.2 cm/s at 40 weeks. The SVC PIV did not change significantly with gestational age and remained stable at 1.50 from 20 to 40 gestational weeks (Fig. 2). We found a significant positive correlation of SVC S-velocity, D-velocity, TAMxV and TAMeanV with fetal head circumference (R = 0.32 to 0.56; *P* < 0.0001) and estimated fetal weight (R = 0.34 to 0.59; P < 0.0001). The A-velocity had a weaker but significant correlation with head circumference (R = 0.13; *P* = 0.003), but no significant correlation with estimated fetal weight (R = 0.075; *P* = 0.091). However, the SVC PIV did not correlate significantly either with fetal head circumference (R = -0.044; *P* = 0.337) or with estimated fetal weight (R = 0.013; *P* = 0.765). Similary, we found no significant correlation between SVC PIV and MCA PIV (R = 0.09; *P* = 0.090). However, we found a significant correlation between SVC TAMxV and MCA TAMxV (R = 0.474; P = < 0.0001), and SVC S-velocity with MCA peak systolic velocity (R = 0.497; *P* < 0.0001).

## Discussion

### Principal findings

This study provides longitudinal reference intervals for Doppler-derived fetal SVC blood flow velocities and PIV for the second half of normal pregnancy. All velocities increased significantly with gestational age, whereas the PIV remained stable throughout 20–40 weeks of gestation and it did not correlate significantly with MCA PI.

### Interpretation of results

Data on human fetal SVC Doppler blood flow velocity measurements are scarce. Previous studies have mainly focused on the diagnosis of fetal arrhythmias using simultaneous recording of SVC and ascending aorta Doppler velocity waveforms to assess atrioventricular and ventriculoartrial intervals of the cardiac cycle [23]. Although two studies [14, 15] reported velocity measurements of SVC blood flow, none had had the aim and design required for establishing reference ranges for these velocities or PIV. When comparing, we note a similar gestational age associated increase in SVC blood flow velocities during the second half of pregnancy in the report published by Nyberg et al. who studied the peak systolic velocity and time-averaged maximum velocity at 3 different gestational age windows (22–26, 28–32 and 36 weeks) during the second half of pregnancy [15].

The SVC PIV did not change signicantly as both the systolic and end-diastolic velocities as well as the TAMxV increased proportionately during 20–40 weeks of gestation. In a previous study by Fouron et al., the SVC S-velocity was reported to be slightly higher and A-velocity deflection during arterial contraction insignificantly deeper in fetuses with absent umbilical artery end-diastolic flow (*n* = 11) compared with normal controls (*n* = 15) [14]. This suggests an increased wave amplitude typically caused by increased inotropic drive of the fetal heart during experimentally imposed hypoxemia [24]. This is also expected to be traceable in SVC PIV as similar velocity changes have been observed in a study of fetal internal jugular vein Doppler in normal and growth restricted fetuses [25]. We found a significant correlation between SVC TAMxV and MCA TAMxV, and SVC S-velocity with MCA peak systolic velocity. This is plausible as these velocities are likely to reflect cerebral blood flow volume. As the fetal cerebral circulation is sensitive to alterations in fetal oxygenation, the recorded mean blood velocities in the SVC (i.e. TAMxV and TAMeanV) may increase as a marker of increased blood flow. We speculate this could be an early sign for clinical use. Furthermore, integrating SVC velocimetry with diameter measurement, volume blood flow representing venous return from the fetal head and upper body can be calculated [15].

### Strengths and limitations

One of the strengths of our study is its longitudinal design. Longitudinal studies are preferred for studying serial changes in physiological parameters with advancing gestation compared with cross-sectional studies. Besides, they require only about a half to a third of the sample size needed in a cross-sectional study to estimate the gestational age specific percentiles with the same precision [26]. Furrthermore, the longitudinal design reflects true individual developmental change during pregnancy, which is important for clinical monitoring using serial observations. We had an adequate sample size as well as enough number of observations per gestational week in the second half of pregnancy (except for the gestational week 40 and above) to be able to calculate reference percentiles with good precision as illustrated in Fig. 2.

The quality of Doppler blood flow veocimetry is operator dependent, and not reporting intra- and inter-observer variability could be considered as a limitation of our study. However, acceptable reproducibility of SVC velocity measurements has been documented previously [15]. One may question whether the variation related to the three experienced operators in our study reduces the possibility for general use of the references, and we acknowledge that they may not reflect the magnitude of variation found around the world. On the other hand, we believe that operators should gain a similar level of skill to optimize diagnostic performance.

Considering that the pulsatile precordial veins tend to have a partially blunted rather than a parabolic spatial blood flow velocity profile [15], our results of SVC TAMeanV were lower than expected. Compared with TAMxV, which depends on the maximum velocity tracing at all times during a cardiac cycle, the TAMeanV is including all pixels recorded and therefore sensitive to over-representation of low velocities along the zero-line (e.g. due to clutter and wall movements) that easily causes underestimation of the pixel intensity-weighted mean. Our study results therefore support the use of TAMxV rather than TAMeanV, particularly when calculating volume blood flow [15, 17].

Our study population was relatively homogenous and consisted mainly of White European women. As fetal growth and size vary significantly in different societies [27] and blood flow is linked to size, we recommend caution when applying and interpreting the reference ranges in other populations. Our results underscore this point showing that SVC Doppler velocities are significantly related to fetal head circumference and estimated fetal weight.

## Conclusion

Longitudinal reference intervals of SVC blood flow velocities and PIV were established for the second half of pregnancy. The SVC velocities increased with advancing gestation, while the PIV remained stable from 20 weeks to term. We believe these measurements have a role in the fetal assessment as they represent the fetal brain circulation.

## Data Availability

The dataset generated and analyzed in the current study is not publicly available. However, anonymized data can be available from the corresponding author on reasonable request.

## Abbreviations

IVC: Inferior vena cava
MCA: Middle cerebral artery
PIV: Pulsatiulity index for vein
SVC: Superior vena cava
TAMeanV: Time-averaged intensity-weighted mean velocity
TAMxV: Time-averaged maximum velocity
